# Antifungal effect of titanium oxide nanoparticles on *Candida glabrata* internalized in human macrophages

**DOI:** 10.3389/fcimb.2025.1714083

**Published:** 2025-10-31

**Authors:** María Elena Gómez-Hernández, Shantal Lizbeth Baltierra-Uribe, Juan Castillo-Cruz, Ricardo Mondragón-Flores, Sirenia González-Pozos, Mónica Araceli Vidales-Hurtado, Blanca Estela García-Pérez

**Affiliations:** ^1^ Instituto Politécnico Nacional, Centro de Investigación en Ciencia Aplicada y Tecnología Avanzada, Unidad Querétaro, Santiago de Querétaro, Mexico; ^2^ Instituto Politécnico Nacional, Escuela Nacional de Ciencias Biológicas, Departamento de Microbiología, Ciudad de México, Mexico; ^3^ Centro de Investigación y Estudios Avanzados del Instituto Politécnico Nacional (CINVESTAV), Departamento de Bioquímica, Ciudad de México, Mexico; ^4^ Centro de Investigación y Estudios Avanzados del Instituto Politécnico Nacional (CINVESTAV), Unidad de Microscopía Electrónica, LaNSE, Ciudad de México, Mexico

**Keywords:** *Candida glabrata*, TiO2 nanoparticles, ROS production, antifungal effect, autophagy, macrophages, photocatalysis

## Abstract

**Background:**

*Candida glabrata (Nakaseomyces glabrata)* is an emergent human fungal opportunist pathogen. Recently, it was considered by the WHO as a fungal priority pathogen due to the high percentage of mortality (20-50%) from invasive candidiasis. *C. glabrata* shows high resistance to azoles, and in recent years, echinocandin resistance has been rising. Additionally, *C. glabrata* can evade immunological responses and manipulate macrophage activity to survive within these immune cells successfully. Titanium oxide (TiO_2_) is a photocatalytic material with recognized antimicrobial properties with and without photoactivation. This study aimed to investigate the antifungal effect of TiO_2_ nanoparticles on the planktonic form of *C. glabrata* and on internalized *C. glabrata* in macrophages.

**Methods:**

TiO_2_ nanoparticles were synthesized by the *sol-gel* method and characterized by X-ray diffraction, Raman spectroscopy, and TEM. The effect of nanoparticles on *C. glabrata* was determined by quantification of Colony Forming Units and TEM. ROS levels were analyzed by NBT reduction, and the role of autophagy was determined by immunofluorescence and western blotting.

**Results:**

The nanoparticles synthetized are composed of a mixture of anatase and rutile phases with a quasi-spherical shape and a diameter range of 10-79 nm. These nanoparticles exhibit a significant effect on planktonic *C. glabrata* (approximately 90%) and a substantial reduction of fungal load (70-90%) in infected macrophages. Remarkably, the lower concentrations (1, 10, and 50 µg/mL) exhibited the best antifungal effect. Moreover, this impairment was associated with independent mechanisms of the ROS production, since at those concentrations, there was no increase in ROS levels, and the antifungal effect was also observed in darkness. The induction of autophagy observed in cells treated with 50 µg/mL nanoparticles suggests that this process is a potential mechanism responsible for TiO_2_ nanoparticles activity.

**Conclusion:**

TiO_2_ nanoparticles exhibited a high antifungal effect on both planktonic and internalized yeasts, suggesting that autophagy may be involved. Together, the findings highlight the potential therapeutic use of TiO_2_ nanoparticles in the treatment of fungal infections.

## 1 Introduction

*Candida glabrata* (previously known as *Nakaseomyces glabrata*) is a significant opportunistic pathogen worldwide. In 2022, *C. glabrata* was classified as a fungal emergent pathogen of high priority by the WHO (2022) due to its high mortality rate (20-50%) and emerging resistance to multiple antifungal agents (Lotfali et al., 2021). In addition, *C. glabrata* is a fascinating microorganism that can manipulate the host immune response to survive and persist, exploiting a wide repertoire of virulence factors. The protease expression, biofilm formation, development of strategies to adapt to a stress microenvironment, and avoidance of the antimicrobial functions of immune cells are some of the virulence factors that *C. glabrata* presents and efficiently utilizes (Castrejón-Jiménez et al., 2022). Macrophages are cells of the innate immune system and are considered professional phagocytes, acting as the first line of defense against invasive pathogens. The protective functions of macrophages are mediated by highly sophisticated mechanisms, including phagocytosis, the hijacking of pathogens in pH-acidic vacuoles, the production of oxygen and nitrogen metabolites, autophagy to target pathogens that escape the phagosome, and cytokine production to modulate the immune response (Shapouri-Moghaddam et al., 2018), among others. These mechanisms act in a coordinated manner to kill pathogens; however, numerous pathogens have developed advanced strategies to subvert the effector functions of macrophages. It is known that *C. glabrata* can counteract the microbicidal mechanisms of macrophages, evading endosomal maturation and phagolysosome fusion (Seider et al., 2011; Kasper et al., 2014). Additionally, *C. glabrata* exhibits high intrinsic resistance to oxidative stress, mediated by several antioxidant defense enzymes, including superoxide dismutases, glutathione reductases, thioredoxins, peroxidases, heat-shock proteins, and enolases (Cuéllar-Cruz et al., 2014). The glucose starvation in the phagosome is overcome by *C. glabrata* by metabolic reprogramming and pexophagy, a specialized form of autophagy (Rai et al., 2012; Roetzer et al., 2011). The extraordinary ability of *C. glabrata* to manipulate the host microbicidal mechanisms and the immune response have facilitated its survival and persistence in macrophages, using them as an intracellular niche and even exploiting them to promote its dissemination and drug resistance (Arastehfar et al., 2023).

The control of intracellular pathogens remains a significant challenge; recently, paradigm shifts for managing intracellular infections have begun to be explored. In this context, renewed research interest has been highlighted in utilizing various nanoparticles as treatment options.

The most common inorganic nanoparticles employed as antibacterial are based on silver, copper, selenium, nickel, zinc oxide, titanium oxide, and magnesium oxide, among others (Ozdal and Gurkok, 2022). Titanium dioxide (TiO_2_) is an inorganic semiconductor compound whose interest in medical applications lies mainly in its photocatalytic activity. Other properties, such as chemical stability in an aqueous environment, affordability, and biocompatibility, increase their potential applications (Querebillo, 2023). There are two crystalline forms of TiO_2_ with photocatalytic activity: anatase and rutile, with band gaps of 3.2 eV and 3 eV, respectively (Blake et al., 1999; Querebillo, 2023). When this material is irradiated with appropriate energy, electron-hole (e-/h+) pairs are generated, which in the presence of water and oxygen, trigger a series of reactions that lead to the formation of reactive oxygen species (ROS) (Guo et al., 2011; Xie et al., 2022). However, this material can catalyze reactions to produce ROS in dark conditions. This effect has been reported in both nanoparticles in suspensions and scaffolds with antibacterial activity (Wiedmer et al., 2016, 2018).

The antibacterial properties of TiO_2_ are mainly due to ROS formation and the subsequent effects of these ROS on several macromolecules of microorganisms (Vatansever et al., 2013). TiO_2_ nanoparticles have attracted attention due to their broad activity against both Gram-positive and Gram-negative bacteria, as well as certain fungi (Serov et al., 2024, Priyanka et al., 2016). The antifungal activities of these nanoparticles have been more extensively explored against *C. albicans*. However, recently other yeasts have been studied such as *C. tropicalis*, *C. parapsilosis*, *C. krusei*, and *C. lusitaniae* (Kermani et al., 2021). Regarding the antifungal effects of nanoparticles against *C. glabrata*, several investigations have been conducted using metallic nanoparticles. For instance, silver nanoparticles have shown effectiveness in reducing biofilm biomass of *C. glabrata* or mixed biofilms of *C. glabrata* and *C. albicans* (Monteiro et al., 2012, 2011; Silva et al., 2013; Bahey et al., 2024). Moreover, selenium nanoparticles have been reported with reduced MIC values compared with conventional antifungal drugs; interestingly, these nanoparticles caused a downregulation of resistance gene expression of *C. albicans* and *C. glabrata* (Hosseini-Bafghi et al., 2022). Bismuth nanoparticles have also been evaluated, although they did not demonstrate significant effectiveness compared to assayed antifungals (Zanganeh et al., 2025). Materials based on TiO_2_ such as polydimethylsiloxane/titanium dioxide (PDMS/TiO_2_) nanocomposite coating have exhibited >50% reduction in *C. glabrata* biofilm formation, even in the dark (Nguyen et al., 2024), and TiO_2_-CeO_2_ nanofibers have also shown inhibition of *C. glabrata* biofilm formation (Firmino et al., 2021). Although research on the antifungal effect of nanoparticles has increased, most studies have focused on inhibiting biofilm formation and yeast growth. The main aim of this work was to evaluate the effect of TiO_2_ nanoparticles on *C. glabrata* viability internalized in macrophages. To our knowledge, this report is the first to investigate the effect of these nanoparticles on the intracellular growth of *C. glabrata*, which could lead to the development of novel therapeutic strategies for controlling fungal infections.

## 2 Materials and methods

### 2.1 Synthesis of TiO_2_ nanoparticles

The TiO_2_ nanoparticles were synthesized by the *sol-gel* method, using as precursors titanium butoxide (Sigma-Aldrich USA, 97%), absolute ethanol (J.T. Baker USA, 40B), deionized water and nitric acid (Sigma-Aldrich USA, 70%), according to a previously reported formulation (Vidales-Hurtado et al., 2021). Briefly, a chemical solution was prepared by mixing titanium butoxide and ethanol (with a molar ratio of 0.019/0.153). Subsequently, water acidified with nitric acid was added (at a molar ratio of 0.149/0.022). This mixture was left to age at room temperature (22-30°C) for one month, until a white *sol* was obtained, indicative of the formation of nanoparticles. The solid phase was centrifuged and, to remove organic residues from the synthesis, two washes with ethanol and one with water were performed. Finally, an aqueous suspension of titanium oxide nanoparticles was obtained.

Diffraction patterns were obtained using a miniflex 600 diffractometer (Rigaku, Tokyo, Japan) with copper radiation. Raman spectra were acquired with a LabRam Confocal Micro-Raman (Horiba Jobin Yvon, Germany) coupled with an OLYMPUS BX41 microscope, using an excitation wavelength of 633 nm. For both measurements, the nanoparticles were previously dried at 55°C for 24 hours.

Transmission electron microscopy (TEM) images were obtained with a JEM 1400 transmission electron microscope (JEOL, Ltd., Japan). Nanoparticle suspensions were diluted in deionized water and deposited on grids coated with a polyvinyl formal plastic and visualized directly in the TEM. The size of the TiO_2_ nanoparticles was measured using image J software and applied fitting distribution to data.

### 2.2 Cell line model

The monocytic THP-1 cell line was obtained from American Type Culture Collection and was maintained in Roswell Park Memorial Institute (RPMI) 1640 medium supplemented with 8% fetal bovine serum at 37°C and 5% CO_2_. For the experiments, 3×10^5^ monocytes were differentiated into macrophages for 48 h using 15 ng/mL forbol 12-miristato 13-acetato (PMA) in a 24 well-plate. After the incubation time, the cells were washed with 1 mL of Hanks´ balanced saline solution (HBSS, Sigma-Aldrich H1387).

### 2.3 Yeast strain

For all assays, the CBS138 reference *C. glabrata* strain was used. The Institut Pasteur in France kindly donated this yeast. The yeast strain was maintained in yeast extract peptone dextrose medium (YPD) and was confirmed using CHROMagar. For all experiments, one isolated colony was inoculated in YPD liquid medium and incubated at 37°C to reach logarithmic phase growth (approximately 14 h). Then, the yeast suspension was centrifuged at 5000 rpm for 3 min and washed three times with HBSS. The yeast number was adjusted according to experiments in a Neubauer chamber. In some experiments, the yeast suspension was adjusted to 1 × 10^6^ yeasts/mL and for infection assays, the yeast suspension was prepared at a MOI of 1:1.

### 2.4 Quantification of ROS production

The production of ROS induced by TiO_2_ nanoparticles was evaluated indirectly using the nitro blue tetrazolium (NBT) reduction technique (Wang et al., 2025). In brief, 1 mL suspensions of 1, 10, 50, 100, 500, and 1000 μg/mL TiO_2_ nanoparticles were placed in 24 well-plates. Afterward, nanoparticles were irradiated with light for 60 min, using a white LED strip light placed parallel to the plate at a distance of 5 cm above. In alternative experiments, nanoparticles were UV irradiated, using NIS G15T8 15W Germicidal UV-C Fluorescent Lamp. Subsequently, 500 μL of supernatant were removed, and 100 μL of a 1.6 mg/mL NBT solution (Sigma-Aldrich N6876) were added. The plates were incubated at 37°C for 45 min in the dark. Then, 208 μL of 2M potassium hydroxide (KOH, Sigma-Aldrich 484016) and 184 μL of dimethyl sulfoxide (DMSO, Sigma-Aldrich D4540) were added to dissolve the formazan precipitates. The absorbance was measured at 600 nm in SpectraMax M3 (Molecular Devices, MT 05939, USA).

ROS production in macrophages treated with TiO_2_ nanoparticles was evaluated by the NBT method (Muñoz-Duarte et al., 2016). 3×10^5^ cells/mL were treated with 1, 10, 50, 100, 500, and 1000 μg/mL TiO_2_ nanoparticles for 1 h. Next, treated macrophages were irradiated with light using the system described in the previous point. Macrophages incubated for 30 min with an opsonized-heat-killed *Saccharomyces cerevisiae* suspension were used as a control for ROS production. After incubation, the supernatants were removed and 500 µL of 1.6 mg/mL NBT solution was added. Cells were incubated at 37°C with NBT solution for 45 min in the dark. Afterwards, 216 μL of 2M KOH and 184 μL of DMSO were added to each well. Finally, the absorbance was measured at 600 nm in the SpectraMax M3.

### 2.5 Viability assay of macrophages treated with TiO_2_ nanoparticles

The effect of TiO_2_ nanoparticles on viability of macrophages were carried out mediated 3-(4,5-dimethylthiazol-2-yl)-2,5-diphenyltetrazolium bromide (MTT) assays (Kermani et al., 2021). In brief, monolayers containing 3 x 10^5^ cells were treated with various concentrations of TiO_2_ nanoparticles (1, 10, 50, 100, 500, and 1000 μg/mL) in RPMI-1640 medium supplemented with 8% fetal bovine serum (FBS) for 24 h at 37°C and 5% CO_2_. Cells without treatment were used as a viability control, and cells treated with 30% DMSO were the death control. After treatment, cells were incubated with 100 µl of 5 mg/mL MTT solution (Sigma-Aldrich USA, M2128) for 2 hours. Finally, the formazan precipitates were dissolved with 200 µL DMSO, and the absorbance at 550 nm was measured using a Spectramax M3.

### 2.6 Determination of CFU/mL of *Candida glabrata* treated with TiO_2_ nanoparticles

The effect of TiO_2_ nanoparticles on *C. glabrata* viability was evaluated by the determination of Colony Forming Units (CFU/mL) (Firmino et al., 2021). 1 × 10^6^ yeasts/mL suspension was seeded in 24-well plates and was treated with 1, 10, 50, 100, 500, and 1000 μg/mL TiO_2_ nanoparticles for 60 min in darkness. In parallel, other wells were irradiated with light for 60 min. Afterwards, to determine the CFU, serial dilutions of the untreated and treated samples were plated in YPD agar. Plates were incubated at 37°C for 24 h, and then, colonies were quantified.

### 2.7 Effect of TiO_2_ nanoparticles on internalized *Candida glabrata* in macrophages

Macrophage monolayers were prepared with 3 x 10^5^ cells in 24-well plates and were infected for 1 h with *C. glabrata* suspension at a MOI of 1:1 at 37°C and 5% CO_2_. To eliminate the extracellular yeasts, the monolayers were treated with 2 μg/mL amphotericin B for 1 h, and the supernatants were recovered and plated on YPD agar to confirm that the extracellular yeast was killed. The infected macrophages were treated with 1, 10, 50, 100, 500, and 1000 μg/mL TiO_2_ nanoparticles for 1 h at 37°C and 5% CO_2_. Then, the macrophages infected and treated were exposed to light irradiation for 1 h at 37°C to activate the nanoparticles or incubated in darkness as a control. Finally, macrophages were lysed with 500 µL of 0.25% sodium dodecyl sulfate (SDS, Sigma-Aldrich USA, L3771) for 5 min and then neutralized with 500 µL 3% bovine serum albumin (BSA, Sigma-Aldrich, USA). The lysates were diluted and plated on YPD agar, and the plates were incubated overnight to quantify the colonies.

### 2.8 TEM assays

The Transmission Electron Microscopy (TEM) assays were performed using macrophages infected as described previously and treated with 50 µg/mL TiO_2_ nanoparticles. As controls, infected cells without treatment and uninfected and untreated cells were used. Cells were processed according to Araujo-Ruiz et al., 2025. In brief, the cell suspensions were fixed with 2.5% glutaraldehyde for 1 h and thoroughly washed with PBS at room temperature (RT). Subsequently, cells were fixed with 1% osmium tetroxide in PBS for 1 h at 4°C and were washed exhaustively with PBS at RT. Then, they were dehydrated through increasing concentrations of ethanol, embedded in Spurr’s resin (Electron Microscope Sciences, Washingtron, D.C.) and polymerized at 60°C for 72 h. Thin sections were obtained with a Reichert-Jung ultramicrotome (Reichert Jung, Austria), mounted on 200-mesh copper grids and stained with uranyl acetate and lead citrate. Sections were examined using a JEM 1400 transmission electron microscope (JEOL, Ltd., Japan).

### 2.9 Determination of proteins regulating autophagy (LC3 and p62/SQSTM1)

The detection of LC3 protein was performed using western blotting. In brief, monolayers of macrophages were prepared using 3.5 × 10^6^ cells/well. Then, macrophages were treated with 1, 50, and 1000 μg/mL TiO_2_ nanoparticles for 1 h. Parallelly, other monolayers were treated with 1 μg/mL rapamycin for 4 h as a positive control for autophagy and 50 μM chloroquine for 24 h as an inhibitor of autophagy. After treatments, cells were lysed with 150 Ml of RIPA buffer (Cell Signaling) and the quantification of total proteins was determined by the Bradford method. Subsequently, SDS-PAGE was performed using Stain-Free gels (4-20%) (BIO-RAD, Hercules, CA, USA) and separated proteins were transferred to a nitrocellulose membrane (BIO-RAD). For immunodetection, membranes were incubated overnight with anti-LC3A/B (Cell Signaling Technology, Inc., Danvers, MA, USA) at a 1:1000 dilution and secondary anti-rabbit IgG-HRP (Santa Cruz Biotechnology, Dallas, TX, USA) at a 1:5000 dilution for 2 h. Bands were visualized in a ChemiDoc™ Touch Imaging System (BIO-RAD) using Clarity™ Western ECL Substrate reagent (BIO-RAD). The images obtained were analyzed with the Image Lab 5.2.1 software. For densitometric analysis, α/β-tubulin (Cell Signaling) was used to normalize the relative expression of LC3.

The detection of p62/SQSTM1 protein was conducted by immunofluorescence. For this, macrophage monolayers were prepared on sterile glass coverslips by seeding 1 × 10^5^ cells/coverslip. Then, cells were infected with *C. glabrata* suspension and treated for 1 h with TiO_2_ nanoparticles at concentrations of 1, 50, and 1000 µg/mL. As controls, rapamycin (1 µg/mL) was used as an inducer of autophagy, and chloroquine (50 µM) was used as an inhibitor of autophagic flux. Monolayers were fixed with 4% paraformaldehyde for 30 min, washed with PBS, and permeabilized with 0.25% Triton X-100 and 0.1% SDS solutions. After, cells were blocked with 3% bovine serum albumin (BSA) for 30 min. Following three PBS washes, a 1:200 dilution of anti-p62 antibody (Abcam) was added, and the samples were incubated overnight at 4°C. The monolayers were then washed three times with PBS and incubated with a 1:200 dilution of FITC-labeled anti-rabbit secondary antibody (Sigma) for 2 h at room temperature. Finally, samples were washed five times with PBS and mounted on glass slides using Vectashield mounting medium containing DAPI (Vector Laboratories). Images were acquired using a Nikon Ti Eclipse inverted confocal microscope (Nikon Corporation) with NIS-Elements v4.50 software with a 20× dry objective lens (Numerical aperture of 0.8).

### 2.10 Statistical analysis

The graphs and statistical analysis were performed by using the GraphPad Prism Software version 10.0.0 (San Diego, CA, USA). Data represent the mean of two or three independent experiments and standard error was graphed. Data were analyzed with one-way ANOVA or Welch’s ANOVA test according with variance homogeneity. *Post hoc* tests were used according to pairwise comparisons. The differences were considered statistically significant when p < 0.05*, p < 0.01**, p <0.001*** or p<0.0001****.

## 3 Results

### 3.1 Crystalline phases and size of TiO_2_ nanoparticles

**Figure 1:**
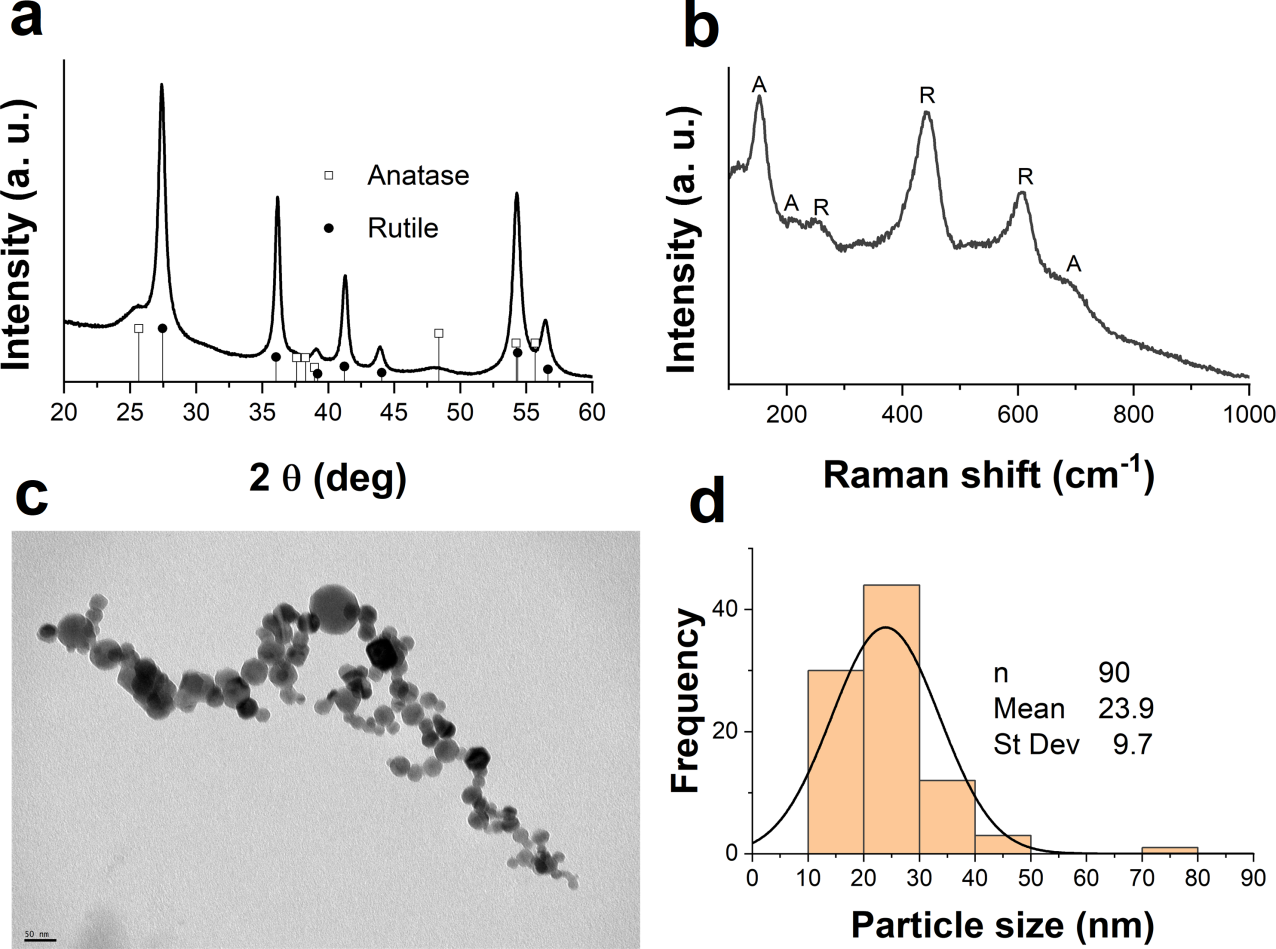
Physicochemical properties of TiO_2_ nanoparticles. **(A)** X-Ray diffraction pattern of TiO_2_ nanoparticles, **(B)** Raman spectra of TiO_2_ nanoparticles, **(C)** Representative transmission electron microscopy image of TiO_2_ nanoparticles and size distribution.

The X-ray result of TiO_2_ nanoparticles (**Figure 1A**) shows that the nanoparticles are a mixture of anatase and rutile phases. The lower intensity peaks at 25.6 (in 2θ scale) correspond to the anatase phase (PDF 2-0406), while the intense peaks at 27.46, 36.05, 41.22, and 54.33 confirm the rutile phase (PDF 4-0551).

This result is consistent with the Raman spectra of **Figure 1B**, where peaks observed are related to both phases. The signs at 152 cm^-1^, 208 cm^-1^, 515 cm^-1^, and the shoulder at 686 cm^-1^ are attributed to anatase phase (Zhang et al., 2000). In fact, the peak at 152 cm^-1^ is a highly sensitive indicator of the presence of this phase (Zhang et al., 2000; Niilisk et al., 2006). On the other hand, the peaks at 439 cm^-1^ and 605 cm^-1^ are related to the rutile phase (Zhang et al., 2000).

**Figures 1C, D** shows TEM images of an agglomerate of the TiO_2_ nanoparticles. The agglomerate is formed during the drying process of particles for analysis. The nanoparticles showed a quasi-spherical shape with a diameter range of approximately 10–79 nm. The aqueous suspension of nanoparticles remained stable for 16 months without signs of precipitation, indicating the dispersion and stability of the nanoparticles in this medium.

### 3.2 TiO_2_ nanoparticles induce the death of planktonic *C. glabrata*

**Figure 2:**
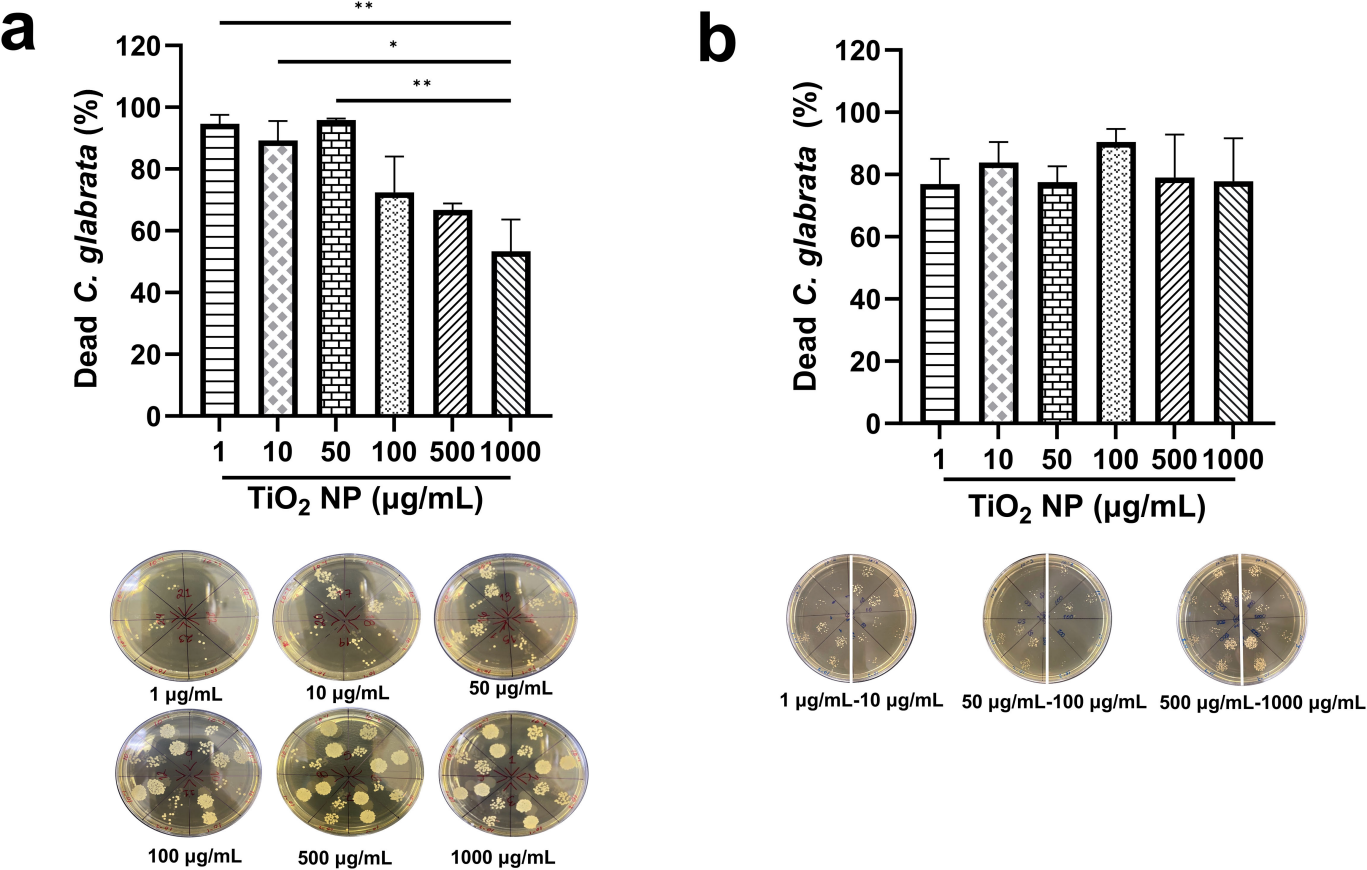
Effect of TiO_2_ nanoparticles on planktonic *C glabrata*. **(A)** Irradiated nanoparticles for 60 minutes and **(B)** nanoparticles incubated in darkness for 60 minutes. The death percentage was calculated considering untreated cells as 100% viability. The bars represent the media of two independent experiments. Figure includes representative images of CFU recuperated from yeast suspension treated with TiO_2_ nanoparticles. **(A)** The statistical analysis used is ordinary one-way ANOVA, with a Tukey *post-hoc* test, with a confidence level of 95%, assuming a value of p<0.05 as statistically significant. **(B)** The data were analyzed with a Welch´s ANOVA test, with a confidence level of 95% (p<0.05*, p<0.01**).

As a next step of this work, the biological properties of TiO_2_ nanoparticles, specifically their antifungal properties against *C. glabrata* were determined. For this, planktonic *C. glabrata* suspensions were incubated with several concentrations of TiO_2_ nanoparticles. Then, the viability of the yeasts after exposure to TiO_2_ nanoparticles was evaluated by determining the colony forming units (CFU/mL). In some experiments, the mixture of yeasts/TiO_2_ nanoparticles was irradiated with light for 60 min to photoactivate the nanoparticles and induce the ROS production. At the same time, in alternative experiments, the mix was incubated in darkness as a control. The irradiated nanoparticles caused a significant reduction in *C. glabrata* viability. Remarkably, the better antifungal effect was achieved with lower concentrations (1, 10 and 50 µg/mL) compared to higher concentrations (100, 500 and 1000 µg/mL). The lower concentrations resulted in a reduction of approximately 90% of the yeast load, whereas the high concentrations caused a decrease in *C. glabrata* viability of between 50% and 70% (**Figure 2A**). On the other hand, when TiO_2_ nanoparticles were not photoactivated, they exhibited antifungal activity similar across all concentrations analyzed, with a rate of cytotoxicity ranging from 70% and 90% (**Figure 2B**).

### 3.3 Effect of TiO_2_ nanoparticles on macrophages viability

**Figure 3:**
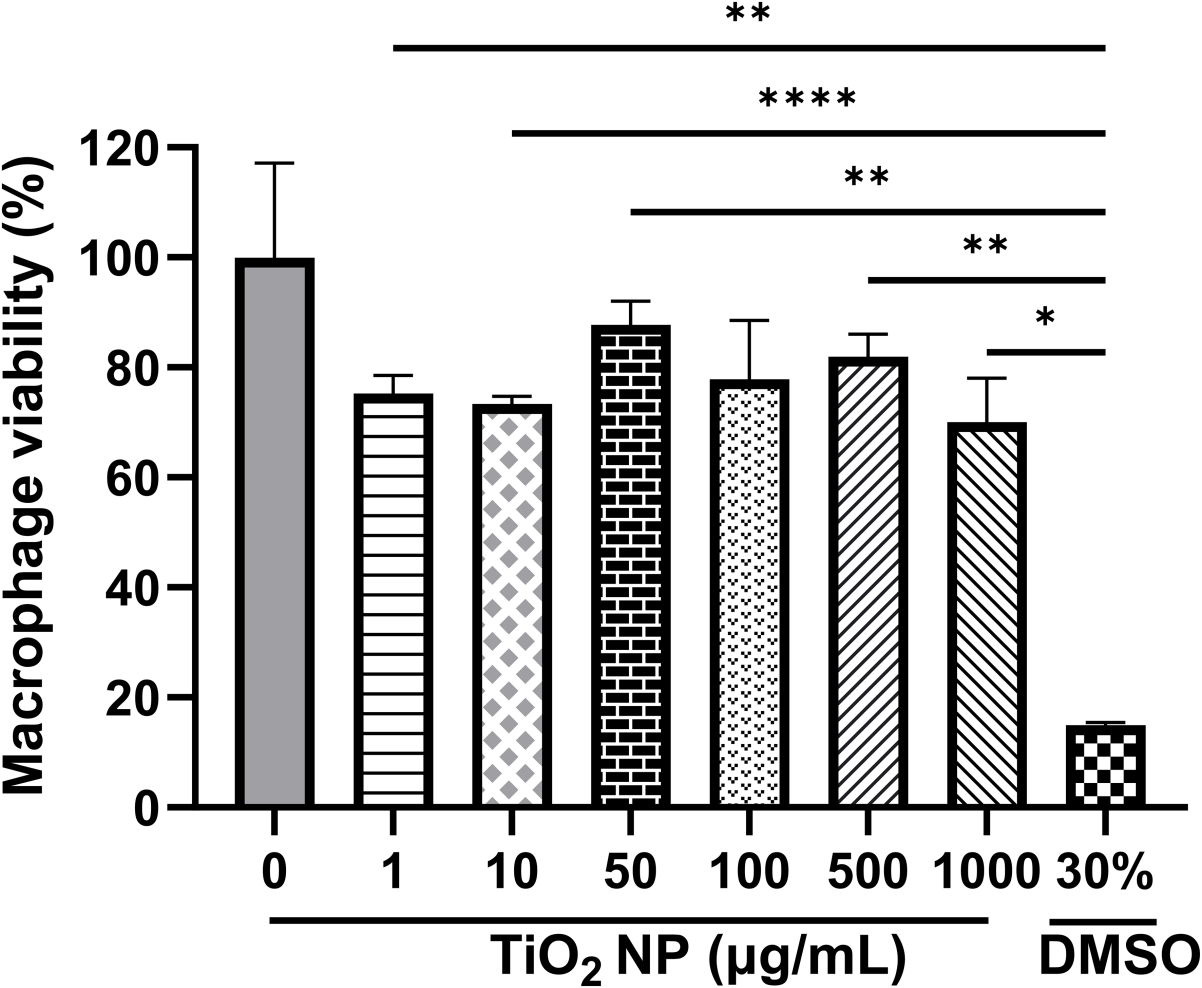
Viability of macrophages treated with TiO_2_ nanoparticles. Cells were treated for 24 hours with 1, 10, 50, 100, 500 and 1000 µg/mL nanoparticles. The percentage viability was estimated considering untreated cells as 100% viability. The bars represent the average of 2 independent experiments with two replicates and SD is shown. Data were analyzed using an ordinary one-way ANOVA test, with a Tukey *post-hoc* test and a 95% confidence level (p<0,05*, p<0.01**, p<0.0001****). Dimetilsulfoxide (DMSO) was used as death control.

The effect of nanoparticles on the viability of macrophages was assessed using the MTT assay. **Figure 3** shows that neither of the nanoparticles’ concentrations tested has a significant effect on the viability of macrophages. All concentrations showed a cell viability percentage close to 80%.

### 3.4 TiO_2_ nanoparticles cause the death of intracellular *C. glabrata*

**Figure 4:**
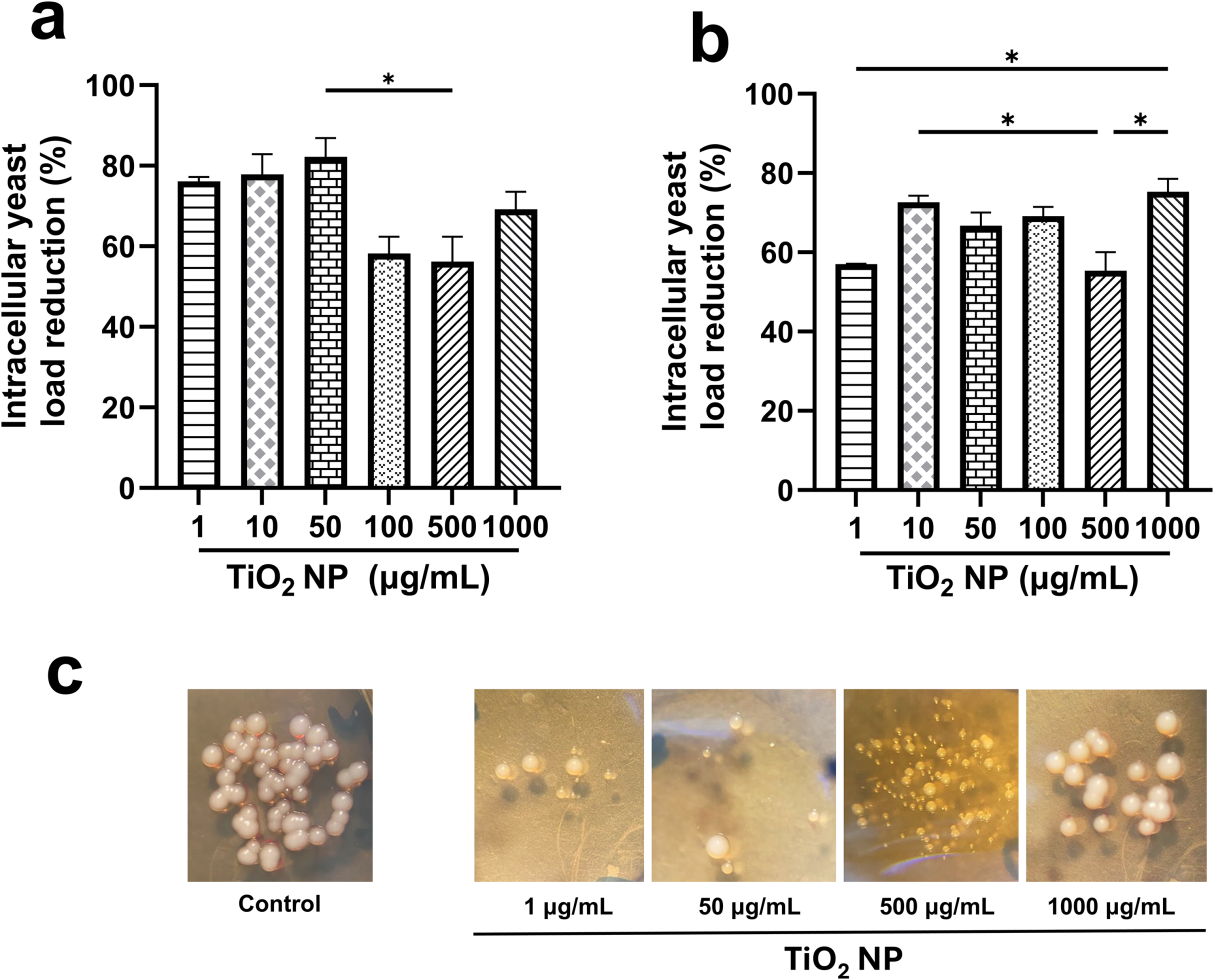
Effect of TiO_2_ nanoparticles on viability of internalized *C glabrata* in macrophages. For this experiment macrophages were infected by 1 h with *C. glabrata* suspension at a MOI of 1:1, then, infected cells were treated with TiO_2_ nanoparticles. **(A)** infected macrophages were treated with TiO_2_ nanoparticles for 1 h and then, irradiated by 1 h **(B)** infected cells were treated with TiO_2_ nanoparticles for 1 h and maintained in darkness for 1 h Reduction of intracellular yeast load was calculated using a control of internalized yeast in macrophages without treatment. Bars represent the average of two independent experiments. The statistical analysis used is ordinary one-way ANOVA, with a Tukey *post-hoc* test, with a confidence level of 95%, assuming a value of p<0.05* as statistically significant.

To evaluate the potential of nanoparticles to kill intracellular *C. glabrata*, macrophages were infected with *C. glabrata* for 1 h at a MOI 1:1. Then, they were treated with several concentrations of TiO_2_ nanoparticles. Two conditions (irradiated by 1 h and non-irradiated) were analyzed to determine the influence of photoactivation on the antifungal activity. The results revealed that the lower TiO_2_ nanoparticle concentrations (1, 10 and 50 µg/mL) also showed a better effect on intracellular yeasts, causing a reduction of 76, 77 and 82% of *C. glabrata* viability whereas the concentrations of 100, 500, and 1000 µg/mL resulting in 58, 56 and 69% of intracellular yeasts reduction respectively (**Figure 4A**). However, this effect was only valid for TiO_2_ nanoparticles irradiated. When nanoparticles were not irradiated, they exhibited different activity (**Figure 4B**) with no clear distinction between the effect showed by lower and higher concentrations; however, they maintained their effect on *C. glabrata*. Notably, the colony morphology of yeasts recovered from lysates of macrophages treated with nanoparticles underwent visible changes, except those recovered from macrophages treated with 1000 µg/mL TiO_2_ nanoparticles, which present morphological characteristics similar to the control. The decrease in size and appearance were the more relevant changes (**Figure 4C**).

### 3.5 Ultrastructural changes of macrophages infected by *C. glabrata* and treated with TiO_2_ nanoparticles

**Figure 5:**
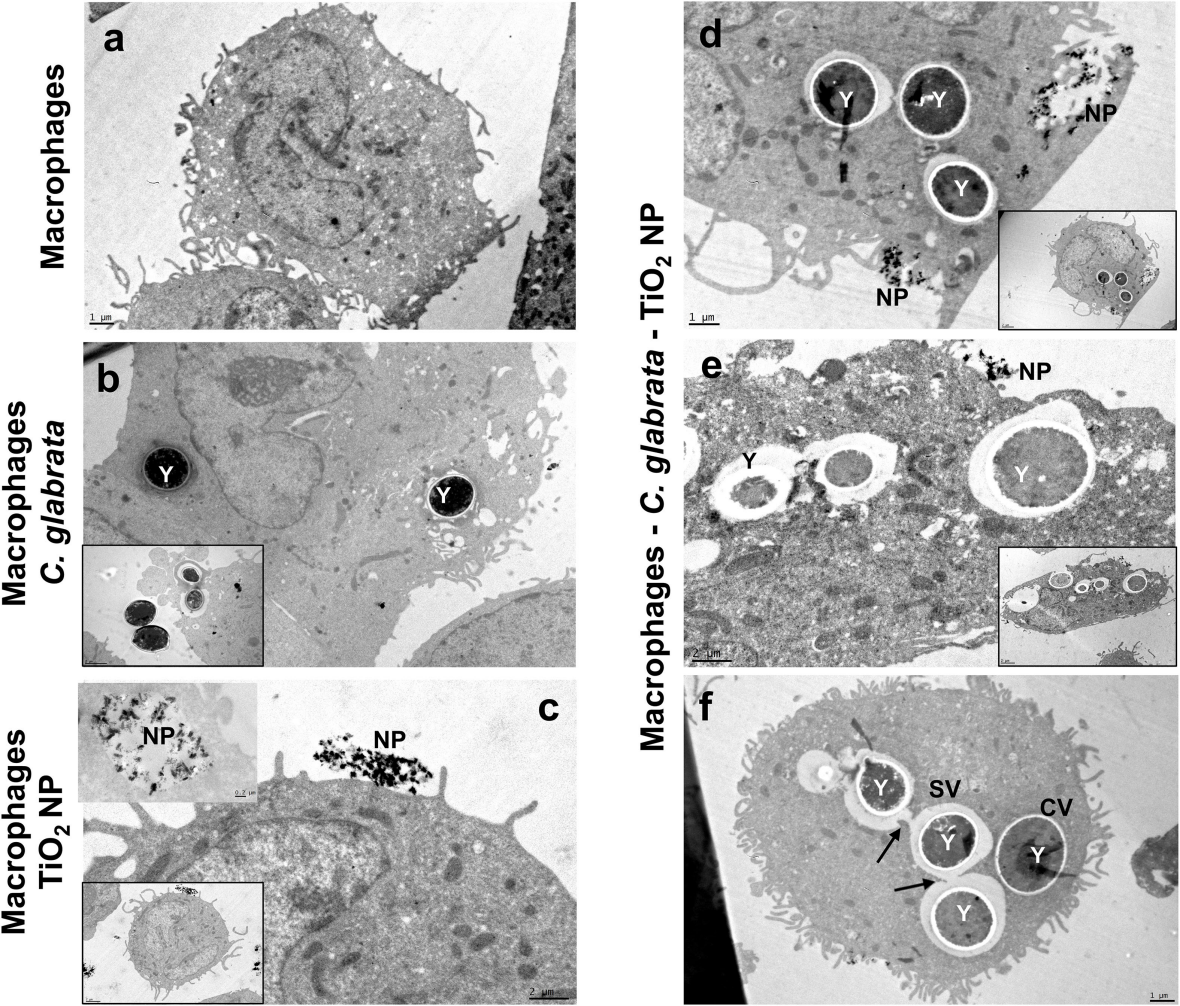
Ultrastructure of macrophages infected by *C glabrata* and treated with TiO_2_ nanoparticles. After infection, macrophages were treated with 50 µg/mL TiO_2_ nanoparticles. **(A)** macrophages uninfected and untreated. **(B)** macrophages infected by *C glabrata* suspension at a MOI of 1:1; intracellular yeasts (Y) are visible in the cytoplasm of the cells. **(C)** TiO_2_ nanoparticles treated macrophages; nanoparticles (NP) were found attached to cytoplasmic membranes and within vacuoles in the cell cytoplasm. **(D–F)** infected and TiO_2_ nanoparticles treated macrophages; images show macrophages with internalized yeasts (Y) and nanoparticles (NP). Some yeasts are contained in closed vacuoles (CV) and others in spacious vacuoles (SV). The fusion of phagosomes is indicated with black arrows.

Ultrastructural analysis showed the classic morphology of macrophages (**Figure 5**). Uninfected and untreated cells exhibit a round shape with numerous short cellular projections and reduced number of mitochondria distributed in cytoplasm. When cells were infected by *C. glabrata*, classic pseudopods were visible to catch closed yeasts. Surprising, internalized yeasts were detectable in tightened vacuoles and spacious vacuoles. TiO_2_ nanoparticle treated macrophages did not show significant ultrastructural changes. Agglomerates of nanoparticles were identified near to cytoplasmic membrane. In contrast, when cells were infected and treated with TiO_2_ nanoparticles showed an increased number of mitochondria close to internalized yeasts. In some cells the presence of nanoparticles around cells was visible. Also, internalized yeasts in various cells showed a significant deleterious changes suggesting the death of them. Unexpected, fusion of spacious phagosomes containing yeasts was evident in cells treated with nanoparticles.

### 3.6 Production of reactive oxygen species induced by photoactivation of TiO_2_ nanoparticles

**Figure 6:**
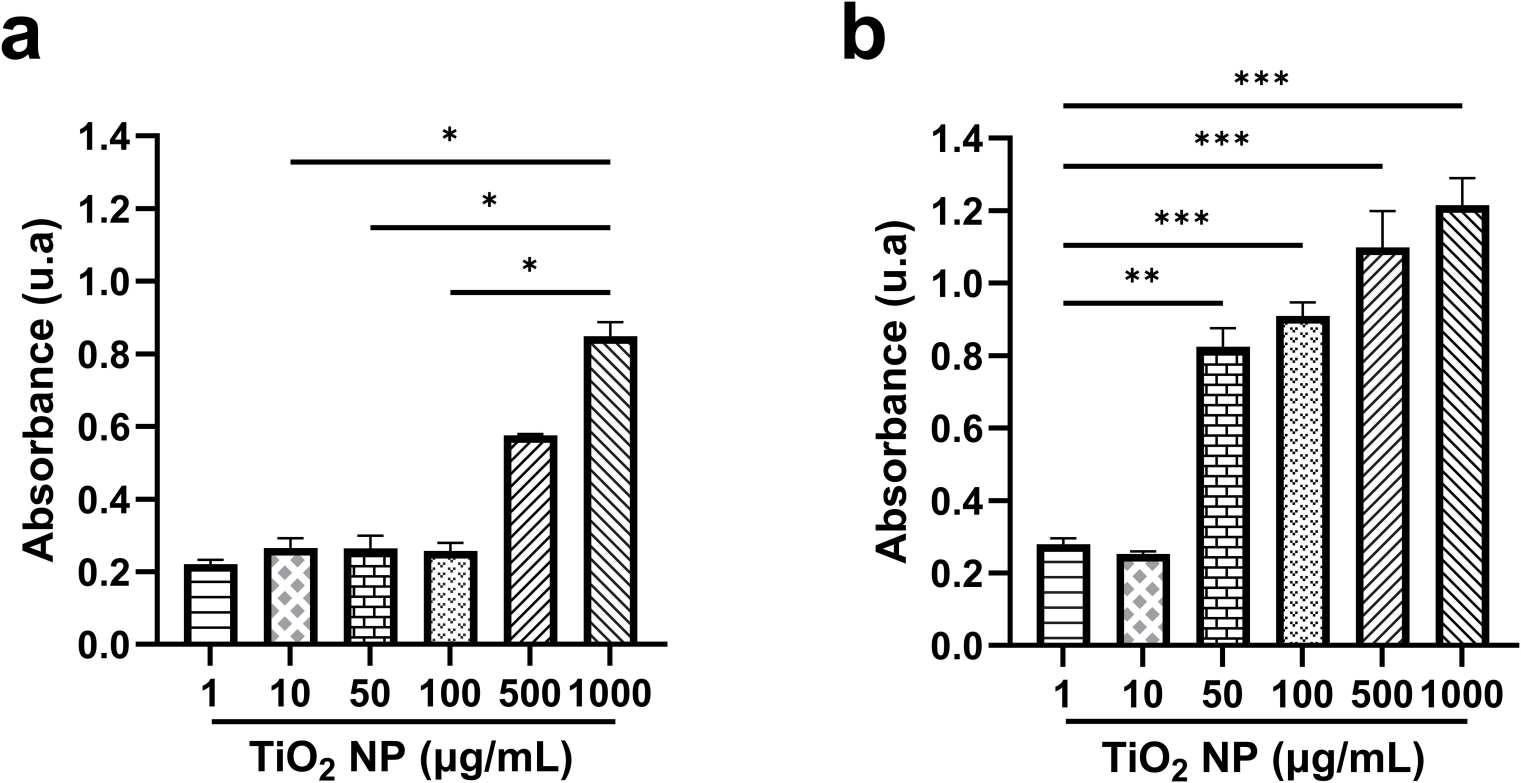
Production of Reactive Oxygen Species (ROS) induced by photoactivation of TiO_2_ nanoparticles. **(A)** levels of ROS after 60 min of activation with light, **(B)** levels of ROS after 60 min of UV irradiation. Bars represent the average of three independent experiments. **(A)** Data were analyzed using Welch’s ANOVA test, with Dunnett’s T3 *post-hoc* test and a 95% confidence level, (p<0.05*). **(B)** The statistical analysis used is ordinary one-way ANOVA, with a Dunnett *post-hoc* test, with a confidence level of 95% (p<0.01**, p<0.001***).

To explore a potential responsible mechanism for the death of the yeasts after exposure to TiO_2_ nanoparticles, the ROS production was determined based on the understanding of the photocatalytic properties of these nanoparticles. First, the ROS production was evaluated after the photoactivation of TiO_2_ nanoparticles for 60 min with light. In this condition the maximum level of ROS production was observed after photoactivation of the higher TiO_2_ nanoparticle concentrations (500 and 1000 µg/mL) (**Figure 6A**). Given the UV absorption of TiO_2_ nanoparticles, the production of ROS was also evaluated after 60 min of UV irradiation. UV photoactivation induced higher ROS production from 50 µg/mL, showing a dose-dependent pattern (**Figure 6B**).

### 3.7 ROS production in macrophages treated with TiO_2_ nanoparticles

**Figure 7:**
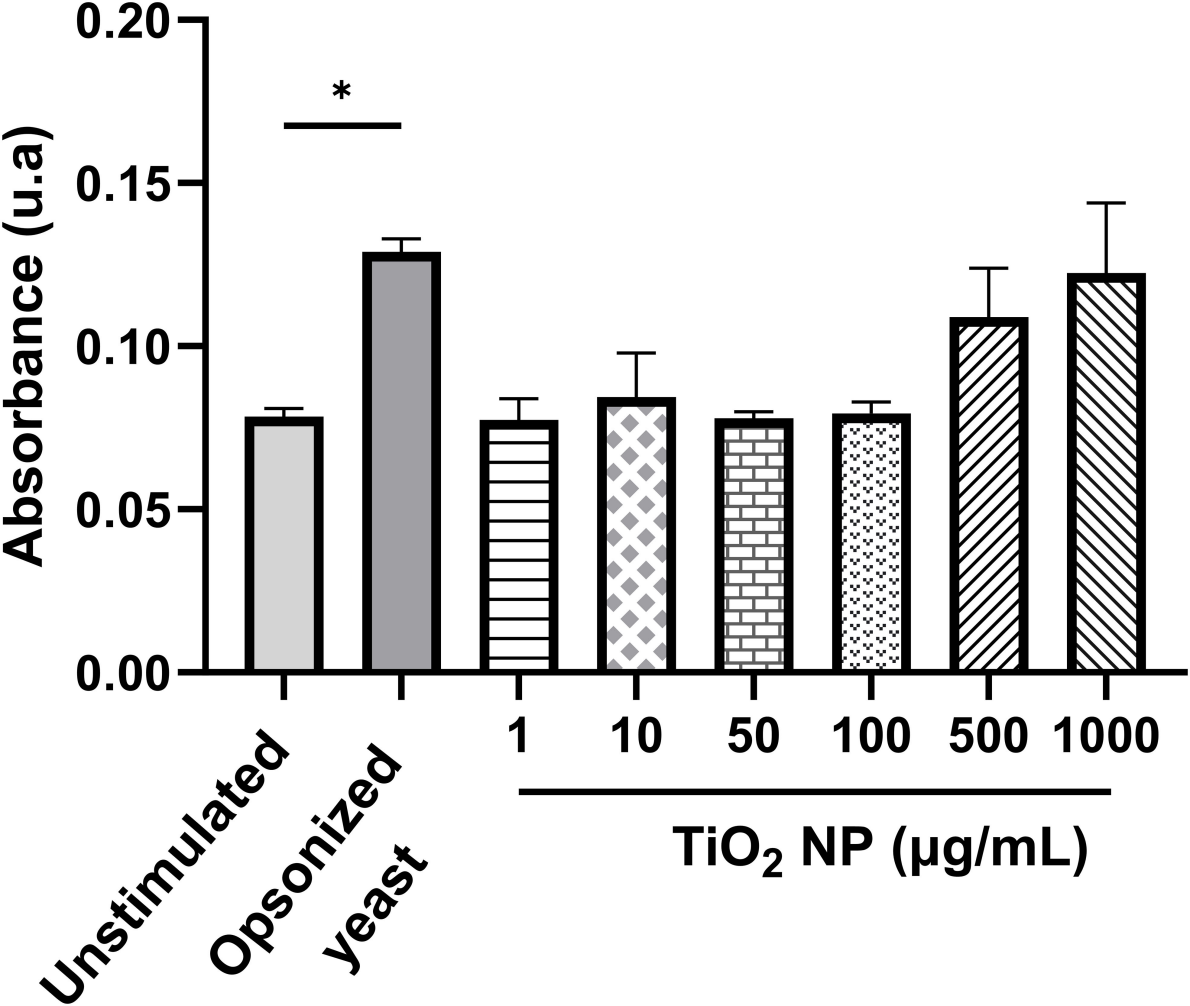
Production of ROS in macrophages treated with TiO_2_ nanoparticles after photoactivation. TiO_2_ nanoparticles treated macrophages were exposed to light by 60 min to photoactivate the nanoparticles. As positive control of ROS production, macrophages were incubated with a suspension of opsonized-heat-killed *Saccharomyces cerevisiae* (Opsonized yeast) for 30 min. Bars represent the average of three independent experiments. The statistical analysis used is Welch’s ANOVA test, with a Dunnett’s T3 *post-hoc*, with a confidence level of 95% (p<0.05*).

On the other hand, experiments were conducted to determine whether TiO_2_ nanoparticles interiorized in macrophages produce ROS after photoactivation. Surprisingly, the macrophages treated with TiO_2_ nanoparticles and irradiated with light did not show a significant increase in ROS production (**Figure 7**).

### 3.8 TiO_2_ nanoparticles induce autophagy in macrophages infected by *C. glabrata*

**Figure 8:**
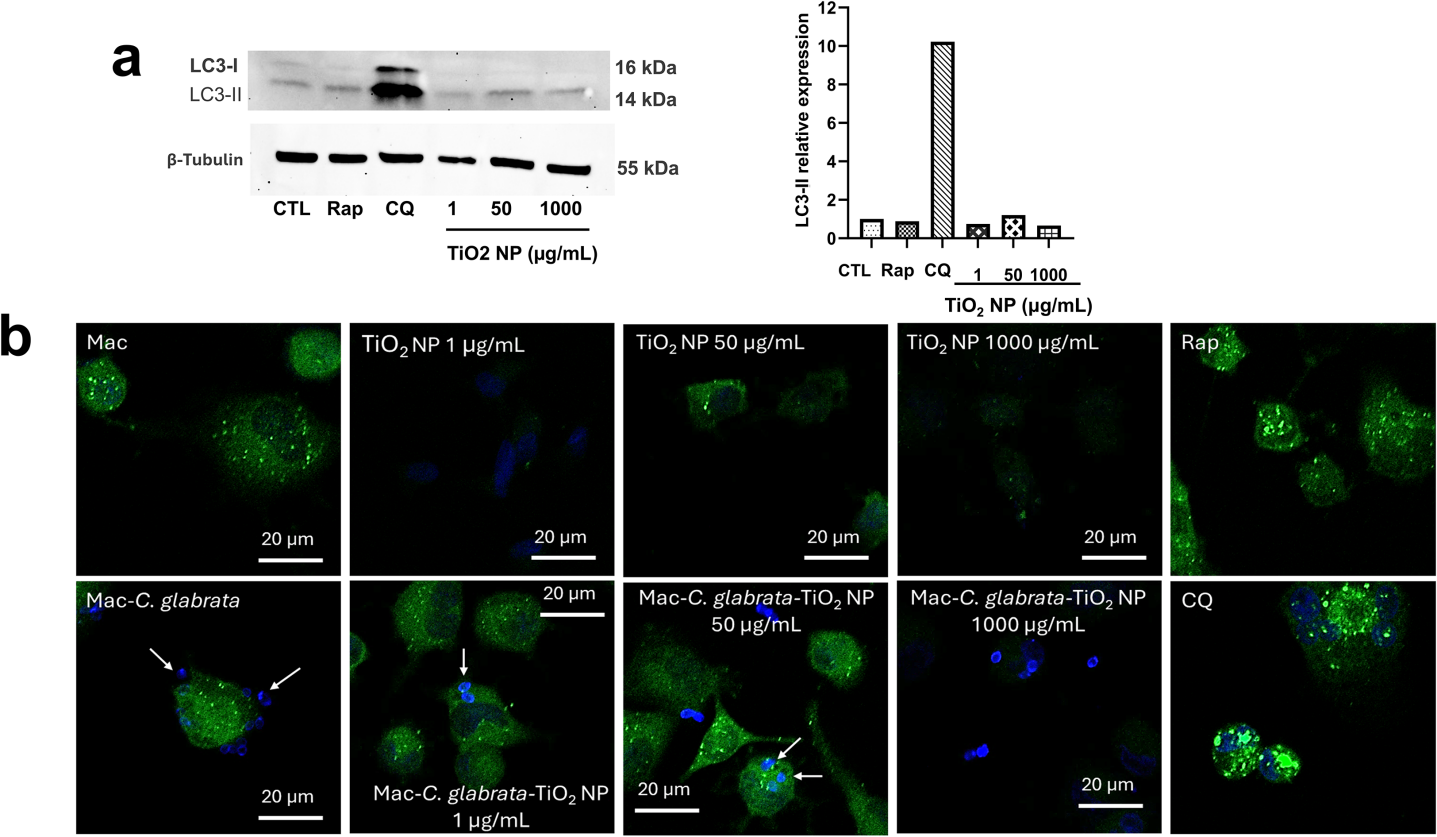
Induction of autophagy in macrophages treated with TiO_2_ nanoparticles. **(A)** Relative LC3-II expression in cells treated with TiO_2_ nanoparticles. The plots show the results of densitometry analyses of representative blots. **(B)** Representative confocal images of the expression of p62/SQSTM1 protein in macrophages treated with TiO_2_ nanoparticles, and macrophages infected by *C glabrata* and treated with TiO_2_ nanoparticles. p62/SQSTM1 protein was labelled with secondary-antibody-FITC (green), nuclei were stained with DAPI (blue), and yeasts were stained with Uvitex 2B (blue). Arrows show yeasts in macrophages. CTL: control, untreated and uninfected cells, Mac: macrophages, Rap: rapamycin.

The previous experiments highlighted that the reduction of intracellular yeasts was independent of ROS production. Therefore, TiO_2_ nanoparticles could trigger other antimicrobial mechanisms in macrophages. Autophagy is a catabolic process that cells can exploit to eliminate cargoes through the lysosomal pathway. It is well known that an array of intracellular pathogens is hijacked and eliminated by this process. We previously reported that autophagy induced in epithelial cells is the mechanism responsible for the death of intracellular *C. glabrata* (Castillo-Cruz et al., 2025). In this context, the potential of TiO_2_ nanoparticles to induce autophagy was evaluated. For that, LC3 protein changes (autophagy marker) were identified in macrophages treated with TiO_2_ nanoparticles. An increase in the expression of LC3-II, indicative of autophagy, was more evident in macrophages treated with 50 µg/mL TiO_2_ nanoparticles (**Figure 8A**). To confirm this finding, the expression of p62/SQSTM1, an adaptor protein of autophagy, was examined. The expression of this protein was also visible in macrophages treated with 50 µg/mL TiO_2_ nanoparticles, but the label was importantly increased in cells infected and treated, which indicate the autophagy activation (**Figure 8B**).

## 4 Discussion

In recent years, infections caused by fungal pathogens have drawn medical attention due to their morbidity and mortality in hospitalized patients and the immunocompromised population. Moreover, the drug resistance acquired by these pathogens increases the difficulty of treating them. Thus, there is a great demand for new alternatives for treatment. Titanium dioxide nanomaterials have attracted attention, and numerous studies have reported their antimicrobial properties. The antifungal effect of TiO_2_ nanoparticles has been more explored against filamentous fungi and yeasts, such as *C. albicans* (Anbumani et al., 2022; Mohammed et al., 2021; Kermani et al., 2021; Peiris et al., 2018). In contrast, the activity of these nanoparticles against *C. glabrata* has been little studied. In this context, this work was designed to investigate the effect of TiO_2_ nanoparticles on planktonic *C. glabrata* and more significantly, on yeast contained within human macrophages. The results clearly revealed that TiO_2_ nanoparticles exhibit high antifungal activity against planktonic yeasts. Notably, the lower concentrations (1, 10, and 50 µg/mL) showed a superior effect compared to the higher concentrations (100, 500, and 1000 µg/mL). It is well established that the morphology (size and shape) and crystal structure of nanoparticles significantly influence their biological properties. The decrease in its size increases the ratio of surface area to/volume, which is related to improving its biological activity (Laroui et al., 2011). On the other hand, amorphous nanoparticles exhibit better antimicrobial effects, followed by those with polygonal and spherical shapes (Serov et al., 2024). In this study, the valuable effectiveness of TiO_2_ nanoparticles against *C. glabrata* correlates with the small size of the nanoparticles (10–79 nm) and their quasi-spherical shape, in addition to their crystal structure. Similar findings have also been reported against other *Candida* species (Kermani et al., 2021). Instead, the minor antifungal effect obtained with the higher nanoparticle concentrations may be due to the formation of aggregates similar to those described for *C. albicans*, where the nanoparticle aggregates and the larger nanoparticles resulted in a poor interaction between nanoparticle-yeast and a decrease in the capacity of nanoparticles to penetrate the yeast wall (Ahmad et al., 2019). For TiO_2_ nanoparticles, photocatalysis is the mechanism that is more described and studied as responsible for their antimicrobial action, due to the oxidative action on the cell wall or cytoplasmic membrane of the microorganisms (Kiwi and Nadtochenko, 2005). However, in this work, the antifungal activity was not entirely dependent on photoactivation, as the nanoparticles kept in darkness also showed fungicidal action. Moreover, the production of ROS was scarce at the concentrations with better antifungal activity, indicating the observed effect was not due to direct damage from ROS overproduction. Previous works have shown comparable results; the property of the TiO_2_/PDMS nanocomposite to inhibit the formation of *C. glabrata* biofilm was independent of ROS production and photoactivation, as even in the dark, the nanocomposite exhibited antibiofilm activity (Nguyen et al., 2024). On the other hand, some experiments conducted without the photoactivation of TiO_2_ nanoparticles have also revealed a deleterious effect on other *Candida* species, indicating the internalization of nanoparticles in the cytoplasm and nucleus, as well abnormality in nuclear division with an increase in the nuclei number (Hifney et al., 2022). Further studies are needed to elucidate the potential antifungal mechanisms independent of photocatalysis. Although some advances have been made in bacteria, the understanding of fungal pathogens remains in their infancy.

One of the primary advantages of *C. glabrata* is its ability to establish and persist within a host cell, thereby constructing a protected niche. This successful behavior is attributed to a range of virulence factors, including resistance to oxidative stress, phagosome arrest, and inhibition of phagolysosome fusion, among others (Castrejón-Jiménez et al., 2022). For this reason, the challenge of treatment is achieving the elimination of pathogens that escape the microbicidal arsenal of host cells. With this motivation, this work tested the effectiveness of TiO_2_ nanoparticles in a *C. glabrata*-infected macrophage model. TiO_2_ nanoparticles caused a significant decrease in intracellular fungal load, with lower concentrations being the most effective. Remarkably, the experiment carried out in darkness also showed a potent microbicide action. In this cell system, the direct effect of nanoparticles, the immune response of the macrophages, or interrelated multiple microbicidal mechanisms acting together could explain the intracellular reduction of fungal load. Although in the experiments, the nanoparticles were irradiated after incubation with infected macrophages, no overproduction of ROS was detected. The production of ROS can originate from the photoactivation of nanoparticles or from the activation of macrophages. It is known that *C. glabrata* can inhibit the production of ROS in macrophages (Seider et al., 2011). In these assays, the infection occurred before the treatment of nanoparticles; therefore, the minimal ROS production detected can be due to the downregulation of these metabolites by the intracellular yeasts. These results suggest a mechanism independent of ROS production; furthermore, when the assay was conducted in darkness, the fungicidal effect was maintained. There are no previous reports that have addressed the microbicidal mechanisms of TiO_2_ nanoparticles. independent of ROS, inside mammalian host cells. However, it is known that TiO_2_ nanoparticles, due to their small size, can reach the intracellular spaces in various types of cells (Rihane et al., 2016; Andersson et al., 2011). Moreover, the phagocytic ability of macrophages could also be involved in the uptake of nanoparticles. Here, electron microscopy revealed the presence of nanoparticles in infected macrophages, with some in contact with cytoplasmic membrane and others contained in vacuoles, indicating a potential interaction between nanoparticles and yeast with subsequent triggering of yeasts killing, as reported by Hifney et al. (2022) for planktonic *C. parapsilosis*. Interestingly, the micrographs also revealed the homotypic fusion of phagosomes containing yeasts. This process has been described as responsible for perturbing the degradative functions of phagosomes and is induced by pathogens to facilitate their survival within host cells (Yu et al., 2022). Here, although the nanoparticles achieve a high percentage of fungal load reduction, some yeasts survived suggesting the phagosome fusion could be a response of survivor’s yeasts to microenvironmental stress originating from nanoparticles. A previous report has demonstrated that echinocandin treatment generates intra-macrophage persistent and drug-resistance yeasts (Arastehfar et al., 2023). Together, these findings emphasize the need for extended treatment times or, where appropriate, study multiple doses to dilucidated whether TiO_2_ nanoparticles treatment is a double-edged sword.

It is known that TiO_2_ nanoparticles also elicit a range of responses in mammalian cells, including changes in phagocytic activity and the induction of proinflammatory responses, among others (Huang et al., 2017; Xu et al., 2016). Autophagy is a catabolic process in cells that can be triggered by several stimuli; in some conditions, this process leads to the elimination of intracellular pathogens (Castrejón-Jiménez et al., 2015). With this in mind, we investigate whether TiO_2_ nanoparticles induce autophagy in macrophages, which could eventually contribute to the killing of yeasts. The susceptibility of intracellular *C. glabrata* to autophagy was previously studied in epithelial cells by our group (Castillo-Cruz et al., 2025). The results showed that one of the concentrations of nanoparticles (50 μg/mL) had better effect on *C. glabrata*, causing an increase in LC3-II and the accumulation of p62/SQSTM1, indicating that autophagy was in progress. Analogous findings were reported in murine macrophages (Dai et al., 2018).

To the best of our knowledge, this work explores for the first time the capabilities of TiO_2_ nanoparticles to kill an intracellular fungus, highlighting their potential in therapy against intracellular infections. Therefore, further investigations addressing its biocompatibility and biological safety may be warranted. Currently, there is accumulating contradictory evidence. In this work, the viability of macrophages exposed to TiO_2_ nanoparticles was investigated, which showed no significant effect on these cells, according to Kose et al. (2020). Nevertheless, further studies should be led to evaluate the balance of advantages/disadvantages of these nanoparticles on human health.

## Data Availability

The raw data supporting the conclusions of this article will be made available by the authors, without undue reservation.
